# Proposition of functional examination according vojta’s concept in children with scoliosis

**DOI:** 10.1186/1748-7161-9-S1-O17

**Published:** 2014-12-04

**Authors:** Kinel Edyta, Gajewska Ewa, Surowinska Joanna, Lisinski Przemyslaw

**Affiliations:** 1 Department of Rheumatology and Rehabilitation, Clinic of Rehabilitation, University of Medical Sciences, Poznan, Poland; 2 Department and Clinic of Rheumatology and Rehabilitation, University of Medical Sciences, Poznan, Poland; 3 Polish Association of Vojta Concept Therapists, Warsaw, Poland

## Background

The father of Vojta’s concept was a Czech neurologist prof. Vaclav Vojta. The beginnings of the theory go back to the 70s of the twentieth century. First Vojta’s patients were children with cerebral palsy. It is currently applied to patients of all ages suffering from neuromuscular system disorders. We also noticed positive results of this therapy during the treatment of children with scoliosis.

## Aim

The study is aimed at presenting examples of the functional examination of children over the age of 6 suffering from scoliosis following Vojta’s method.

## Design

Case series.

## Method

Apart from the basic clinical examination before the therapy (and radiological results: Cobb angle, Risset test), each patient undergoes a thorough functional examination. This includes the assessment of supporting-extensory mechanisms in the standing position, all fours position and the supine position. Additionally, the examination involves the Beighton hypermobility score (to determine the degree of joint laxity) and the mobility of temporomandibular joints. In case of detecting supporting-extensory mechanism disorders the recommended starting position for the exercises, according to Vojta's concept, may be crawling or the first position upon activating of the relevant movement trigger zones. Depending on the type of scoliosis the starting positions are subject to individual modifications.

## Results

On the basis of observations involving children with scoliosis and the analysis of the proposed examination scheme before and after the therapy it was concluded that the quality of supporting-extensory mechanisms, which are crucial to correct body posture, balance and coordination, has improved.

## Conclusions

The proposed functional examination according Vojta’ s concept can be useful in the clinical assessment of children with scoliosis both before and after the therapy. Further research needs to be conducted, based on the proposed functional examination, to confirm the efficiency of the method according to the evidence-based medicine principles.

## References

[B1] Vojta V Peters A The Vojta Principle 2007 Springer-Verlag, Berlin Heidelberg

[B2] Beighton P Solomon L Soskolne CL Articular mobility in an African population Ann Rheum Dis 1973 32 413 8 10.1136/ard.32.5.413 4751776 PMC1006136

